# Exploration of some physical properties of new half-Heusler compounds BiXSr (X = Li and K) using first-principles calculations

**DOI:** 10.1039/d5ra09963c

**Published:** 2026-05-11

**Authors:** Junhong Wei, Yongliang Guo, Guangtao Wang

**Affiliations:** a School of Science, Henan Institute of Technology Xinxiang 453003 China weijh@hait.edu.cn ylguo@hait.edu.cn; b School of Physics, Henan Normal University Xinxiang 453007 China wangtao@htu.cn

## Abstract

Good thermoelectric (TE) materials with high energy conversion efficiency are required to improve energy utilization and help meet increasing energy demands. By combining first-principles calculations with the Boltzmann transport theory, this study systematically investigates the electronic structure, mechanical properties, and TE performance of the half-Heusler compounds BiLiSr and BiKSr for the first time. Phonon spectrum calculations indicate that BiXSr (X = Li and K) exhibits dynamic stability. The calculated elastic constants demonstrate that BiXSr (X = Li and K) is mechanically stable and ductile. Electronic structure analysis reveals that BiLiSr is a direct-bandgap semiconductor, whereas BiKSr is an indirect-bandgap semiconductor. The TE performance results for BiXSr (X = Li and K) show that the Seebeck coefficient is superior under p-type doping, whereas the power factor is higher under n-type doping. Under n-type doping conditions, the maximum power factor values for BiLiSr and BiKSr are 852.14 and 572.85 µW m^−1^ K^−2^, respectively. At 300 K, the lattice thermal conductivity of BiKSr is consistent with previous theoretical studies. At 900 K, the calculated electronic TE figure of merit (*ZT*_e_) for both BiLiSr and BiKSr is 0.99. Considering the dynamic stability, mechanical stability, and TE performance of BiXSr (X = Li and K), this series of compounds demonstrate potential as promising TE materials over a wide temperature range.

## Abstract 2

The TE properties, lattice thermal conductivity, electronic thermal conductivity and *ZT*_e_ ratio of BiXSr (X = Li and K) were calculated at different temperatures.

## Graphical abstract

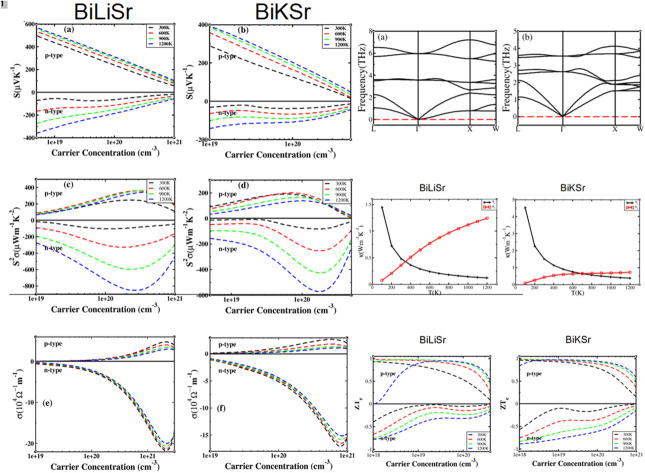

## 1. Introduction

With developments in science and technology, the demand for energy has increased. Therefore, there is an urgent need to develop clean, green, and renewable energy sources to replace non-renewable energy sources and reduce dependence on fossil fuels. Thermoelectric (TE) materials, which convert waste heat into electricity, have attracted significant attention.^1–6^ TE power generation is defined as the conversion of heat energy into electricity through the Seebeck effect.^7,8^

The energy conversion efficiency of TE materials depends on the figure of merit, which is dimensionless, and its mathematical expression is as follows:1
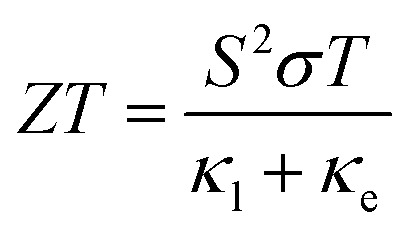
where *S* is the Seebeck coefficient, *σ* is the electrical conductivity, *T* is the absolute temperature, *κ*_l_ is the lattice thermal conductivity, and *κ*_e_ is the electronic thermal conductivity.^9,10^ A high *ZT* value is a criterion for determining good TE materials; thus, achieving a high *ZT* value is very important for TE properties. The power factor (*S*^2^*σ*) and thermal conductivity (*κ* = *κ*_l_ + *κ*_e_) in eqn (1) are interdependent.^3^ Most materials have high thermal and electrical conductivities; therefore, it is difficult to identify a TE material with a high *ZT* value because of its high power factor and low thermal conductivity. Researchers have attempted to identify additional TE materials, such as chalcogenides,^11^ oxides,^12,13^ triple-point metals,^14^ Heusler alloys,^15–18^ and half-Heusler alloys.^19–27^ Half-Heusler compounds have attracted the attention of researchers because of their high Curie temperatures (*T*_C_), good thermal and mechanical stabilities, rich elemental composition, and excellent TE properties at medium and high temperatures. Moreover, their electronic band structures are tunable, and they exhibit better lattice matching with zinc blende semiconductors.^28,29^ Therefore, half-Heusler compounds are promising candidates for spintronic and TE applications.

Gautier *et al.*^30^ reported that 54 out of 400 previously unreported half-Heusler compounds are thermodynamically stable. Similarly, Carrete *et al.*^31^ found that 77 out of 450 half-Heusler compounds are stable. All literature surveys demonstrate that half-Heusler compounds are the perfect choices for TE material applications. Moreover, several half-Heusler compounds have been investigated and developed for various TE applications.^19–24^

Half-Heusler compounds possess good thermal and mechanical stability, rich elemental composition, environmental friendliness, and excellent thermoelectric properties at medium-to-high temperatures, which makes them promising candidates for thermoelectric applications.^28^ Therefore, in the present work, we aim to explore high-performance thermoelectric materials. The selection of the half-Heusler compounds BiXSr (X = Li and K) is mainly motivated by their low thermal conductivity, as reported by Carrete *et al.*^31^ (BiLiSr = 3.04 W m^−1^ K^−1^, BiKSr = 1.96 W m^−1^ K^−1^) and Liu *et al.*^32^ (BiLiSr = 3.0 W m^−1^ K^−1^, BiKSr = 2.0 W m^−1^ K^−1^). Low lattice thermal conductivity is one of the key requirements for excellent thermoelectric materials, indicating that these compounds are promising TE materials. To date, however, BiXSr (X = Li and K) have received little research attention, especially regarding their electronic band structures, phonon transport properties, and underlying microscopic mechanisms. Furthermore, the thermoelectric properties of BiXSr (X = Li and K) based on Boltzmann transport theory have not been sufficiently elucidated. To fill this gap, we systematically investigate the elastic constants, phonon dispersion curves, electronic structures, and thermoelectric transport properties of BiXSr (X = Li and K) using first-principles calculations.

## 2. Methodology

The structural properties of BiXSr (X = Li and K) were obtained using density functional theory (DFT) as implemented in the Vienna *ab initio* simulation package (VASP).^33^ The Perdew–Burke–Ernzerhof generalised gradient approximation (GGA) was adopted for the exchange–correlation potential.^34^ Electron–ion interactions were treated using the projector-augmented plane-wave approach.^35^ A 20 × 20 × 20 *k*-point mesh was used in the Brillouin zone (BZ), and the plane-wave kinetic energy cut-off was set to 600 eV. To fully optimise the geometry of the BiXSr (X = Li and K), the energy convergence threshold was set to 10^−7^ eV, and the force convergence threshold was set to −0.001 eV Å^−1^. The electronic structure and transport properties of the half-Heusler compound BiXSr (X = Li and K) were calculated using DFT with the full-potential linearised augmented plane wave^36^ method, as employed in the WIEN2k code.^37^ The Tran–Blaha modified Becke–Johnson (TB-mBJ) potential was used to accurately predict the bandgap and transport properties.^38,39^

To calculate the TE properties, the semi-classical Boltzmann transport theory within the constant scattering time approximation (CSTA) was implemented using the BoltzTraP code,^40,41^ and the rigid band approximation (RBA) was applied. To ensure precise TE properties, a dense 40 000 *k*-point mesh was used for BZ sampling. The Phonopy code^42^ was used to obtain the phonon spectrum. In the calculations, the supercell was composed of 3 × 3 × 3 cells based on the optimised crystallographic primitive cell. BZ integration was performed using a 2 × 2 × 2 *k*-point mesh. The forces induced by small displacements were calculated using VASP.^33^

The lattice thermal conductivity (*κ*_l_) of BiXSr (X = Li and K) was calculated using both Slack's equation and the Callaway model.^43,44^ Slack's equation is expressed as follows:2
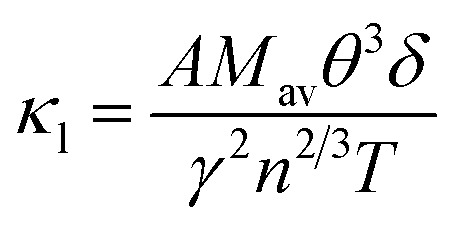
where *M*_av_, *δ*, *n*, *T* and *θ* represent the average atomic weight, the cube root of the average atomic volume, the number of atoms per unit cell, the absolute temperature, and the Debye temperature in Kelvin, respectively. The constant *A* depends on the Grüneisen parameter *γ*, which can be calculated as follows:^45^3
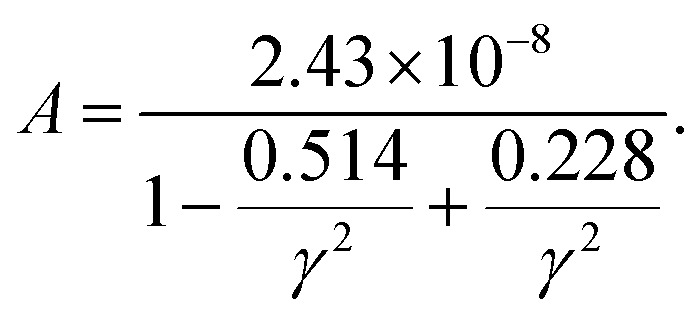


## 3. Results and discussion

### 3.1 Structural and mechanical properties

**Fig. 1:**
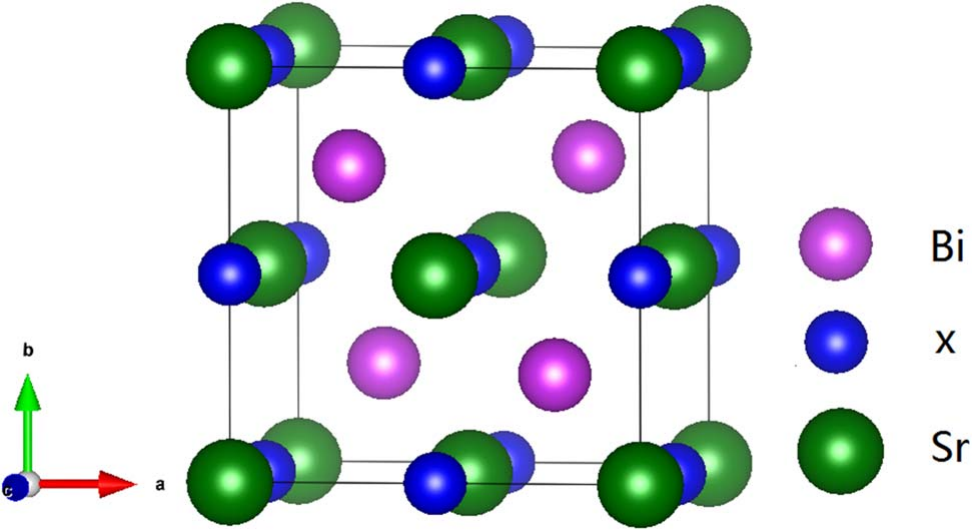
Crystal structure of half-Heusler compounds BiXSr (X = Li and K), where violet spheres represent Bi atoms, blue spheres represent Li or K atoms, and green spheres represent Sr atoms.

Half-Heusler compounds BiXSr (X = Li and K) have a non-centrosymmetric cubic phase structure with the space group *F*4̄3*m* (216).^46^ The structure can be represented as ABC, where the Wyckoff sites are occupied by Bi, X, and Sr elements at the 4c (0.5, 0.5, 0.5), 4b (0.25, 0.25, 0.25), and 4a (0, 0, 0) sites, respectively, as shown in Fig. 1. Using the VASP software package, the lattice constants were first optimised, and the mechanical parameters of the materials were calculated. Table 1 lists the optimised lattice constant (*a*_0_), energy gap (*E*_g_), bulk modulus (*B*), shear modulus (*G*), and elastic constant (*C*_*ij*_) of the half-Heusler compounds BiXSr (X = Li and K). *C*_11_ is an elastic constant that describes the resistance of a material to changes in its longitudinal length during longitudinal loading. *C*_44_ is an elastic constant that describes the resistance of a material to changes in its lateral length during lateral loading.

**Table 1:** Calculated values of the lattice constant (*a*_0_) (Å), bandgap (*E*_g_) (eV), elastic modulus (*C*_11_, *C*_12_, and *C*_44_) (GPa), bulk modulus (*B*) (GPa), shear modulus (*G*) (GPa), Young's modulus (*Y*) (GPa), Poisson's ratio (*ν*), Pugh's ratio (*B*/*G*), average sound velocity (*ν*_m_) (m s^−1^), longitudinal velocity (*ν*_l_) (m s^−1^), shear sound velocity (*ν*_s_) (m s^−1^), and Debye temperature (*Θ*_D_) (K) for BiXSr (X = Li and K)

Parameter	BiLiSr	BiKSr
*a*_0_	8.20	7.56
*E*_g_	1.53	0.99
*C*_11_	40.30	31.43
*C*_12_	15.25	12.52
*C*_44_	4.37	9.09
*B*	23.60	18.82
*Y*	18.55	23.82
*G*	6.77	9.24
*ν*	0.37	0.29
*B*/*G*	3.48	2.04
*γ*	2.45	1.89
*C*_12_ − *C*_44_	10.87	3.43
*ν*_l_	2646.51	2774.32
*ν*_s_	1205.83	1685.54
*ν*_m_	1359.23	1685.54
*Θ*_D_	122.50	140.10

The Born–Huang stability criterion^47^ was used to determine the mechanical stability of the materials, which is expressed as follows:

4 *C*_11_ > 0, *C*_44_ > 0, *C*_11_ − *C*_12_ > 0, and *C*_11_ + 2*C*_12_ > 0,

where *C*_12_ is the elastic constant describing the resistance of the material to changes in its lateral length during longitudinal loading. From Table 1, the calculated elastic constants conformed to the Born–Huang stability criteria; therefore, the BiXSr (X = Li and K) compounds are mechanically stable.

The bulk modulus (*B*) and shear modulus (*G*) were evaluated using Voigt–Reuss–Hill (VRH) approximations.^48,49^ The elastic properties, such as the bulk modulus (*B*), shear modulus (*G*), Young's modulus (*Y*), Poisson's ratio (*ν*), longitudinal velocity (*ν*_l_), transverse velocity (*ν*_s_), and average sound velocity (*ν*_m_) were defined^50^ as follows:5
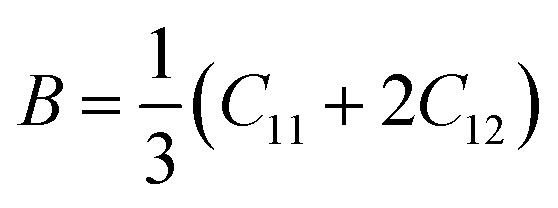
6
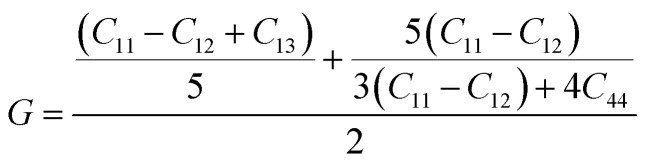
7
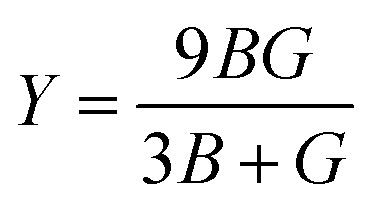
8
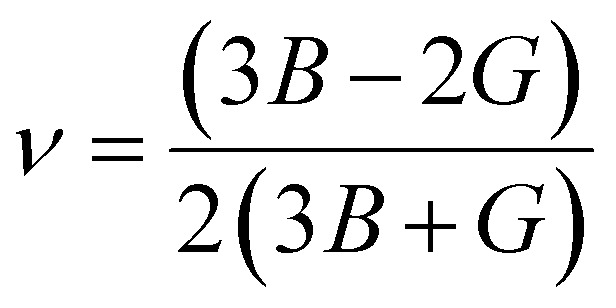
9
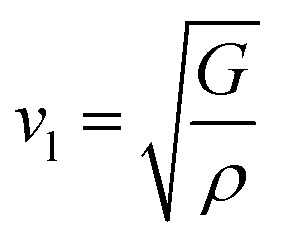
10
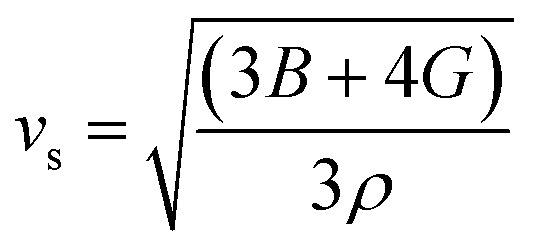
11
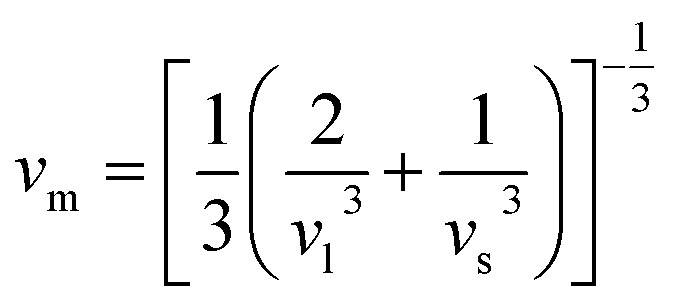


The Debye temperature (*Θ*_D_) was calculated using the Anderson formula,^51^ which is expressed as follows:12
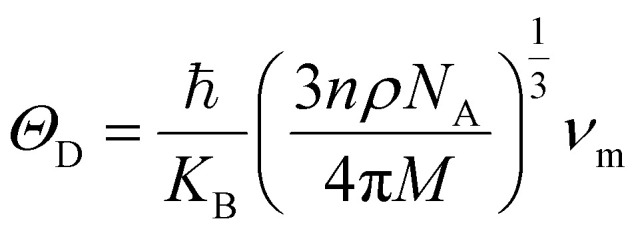
where *K*_B_, ℏ, *n*, *N*_A_, *ρ*, and *M* represent the Boltzmann constant, Planck constant, number of atoms per formula unit, Avogadro's number, density of the unit cell, and atomic mass, respectively. According to the Pugh criteria,^52^ a material is considered ductile if its Pugh ratio (*B*/*G*) is greater than 1.75; otherwise, it is considered brittle. The Pugh ratios of BiLiSr and BiKSr are 3.484 and 2.037, respectively, indicating that both materials are ductile. The Cauchy pressure (*C*_12_ − *C*_44_) also determines the ductility/brittleness of a material: a positive value indicates ductile behaviour, while a negative value corresponds to brittle characteristics.^53^

In addition, fragile materials are not conducive to TE applications,^54^ indicating that BiXSr (X = Li and K) compounds are ductile and suitable as TE materials. If the Poisson's ratio is in the range of 0.25–0.50, the interaction forces between atoms are naturally central.^55^ The calculated Poisson's ratio (*ν*) suggests that the interatomic forces in BiXSr (X = Li and K) are central forces. Based on the resulting Poisson's ratios, the average Grüneisen parameters (*γ*)^56^ of BiXSr (X = Li and K), as listed in Table 1, were calculated as follows:13
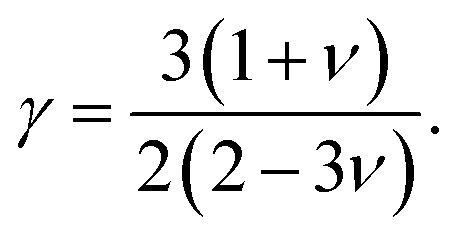


### 3.2 Dynamical stability

**Fig. 2:**
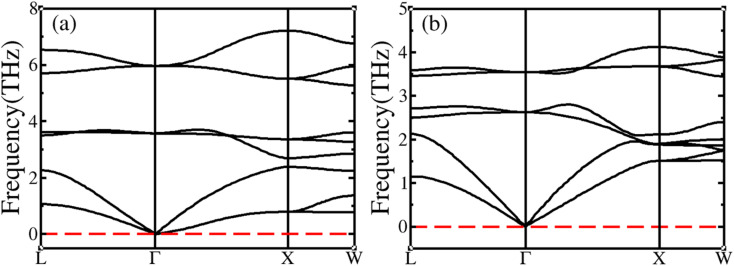
Calculated phonon dispersion curves for (a) BiLiSr and (b) BiKSr.

Phonon scattering plays an important role in understanding the dynamic stability and the optical and thermodynamic properties of materials. To further confirm the dynamic stability of BiXSr (X = Li and K), its phonon spectra were calculated, as shown in Fig. 2. For the phonon dispersion calculations of BiXSr (X = Li and K) compounds, primitive cells were used that contained three atoms and could generate nine vibrational modes, including three acoustic (low-frequency phonon) and six optical (high-frequency phonon) modes. There was no imaginary frequency in any of the dispersion plots; therefore, the BiXSr (X = Li and K) compounds are dynamically stable.

For BiXSr compounds, the acoustic frequency was lower than the optical frequency. The maximum acoustic frequencies of BiLiSr and BiKSr are 2.38 and 2.15 THz, respectively, and the maximum optical frequencies are 7.22 and 4.14 THz, respectively. Although the frequency of the acoustic mode is smaller than that of the optical mode, the contribution of the acoustic mode to the heat transfer is greater than that of the optical mode. This is because the acoustic mode has a large group velocity and high dispersion, whereas the optical mode exhibit a small group velocity and weak dispersion. The intersection of these low-frequency optical modes and high-frequency acoustic modes in the BiXSr compound indicates that the compound exhibits strong phonon–phonon scattering.^57^

### 3.3 Electronic structure properties

**Fig. 3:**
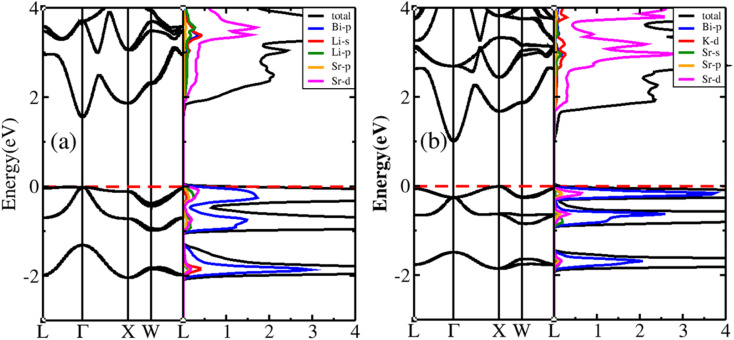
Band structure and PDOS of BiXSr (X = Li and K) for (a) BiLiSr and (b) BiKSr.

Using the optimised lattice constant, the band structures and projected density of states (PDOS) of the BiXSr (X = Li and K) compounds were calculated, as shown in Fig. 3. The conduction band minimum (CBM) and valence band maximum (VBM) of BiLiSr are located at the Γ point in the BZ, while those of BiKSr are located at the X and Γ points, respectively. Therefore, BiLiSr is a direct-bandgap semiconductor, while BiKSr is an indirect-bandgap semiconductor, with bandgaps of 1.53 and 0.98 eV, respectively.

As the atom moves from Li to K, the bandgap of BiXSr decreases. Thus, the Seebeck coefficient of BiLiSr is expected to be slightly higher. In addition, the VBM edge is flat, indicating strongly localised holes, whereas the CBM edge is dispersed, indicating the presence of free electrons. Therefore, the TE performance of p-type BiXSr (X = Li and K) is expected to be superior to that of n-type BiXSr. From the PDOS analysis, it is found that for BiLiSr, in the valence band (VB) region from −2 to −1.3 eV, the density of states is mainly determined by the Bi-p state, with small contributions from Sr-p and Sr-d states. However, in the −1 to 0 eV range, the Bi-p state makes the greatest contribution, while the hybridized states of Li-s, Sr-p, Li-p, and Sr-d contribute less. The density of states above the Fermi level was mainly composed of Sr-d states, whereas the contributions of the Li-p and Li-s states were relatively small.

For BiKSr, from −1.9 to −1.5 eV, the density of states is mainly determined by the Bi-p state, with small contributions from Sr-p and Sr-d states. However, in the −0.92 to 0 eV range, the contribution from the Bi-p state exhibit the largest hybridisation with the Sr-d state, while the contributions from Sr-p and Sr-p states are relatively small. The density of states above the Fermi level is mainly composed of Sr-d states, whereas the contributions from the K-d and Sr-s states are relatively small.

### 3.4 Electron transport properties

Based on the Boltzmann transport theory, the TE properties of BiLiSr and BiKSr, such as the Seebeck coefficient (*S*), electrical conductivity (*σ*), power factor (PF), electronic thermal conductivity (*κ*_e_), and lattice thermal conductivity (*κ*_l_), were calculated at different temperatures with carrier concentrations ranging from 1 × 10^19^ to 1 × 10^21^ cm^−3^, which is an optimum range for good TE performance.^58^ The relaxation time constant (*τ*) depends on the doping level and temperature of the material. To obtain accurate TE parameters for BiXSr (X = Li and K), references were made to XTaZ (X = Pd, Pt and Z = Al, Ga, In)^59^ and NiTiSn.^60^ A constant relaxation time of *τ* = 1 × 10^−15^ s was used throughout the calculations.

**Fig. 4:**
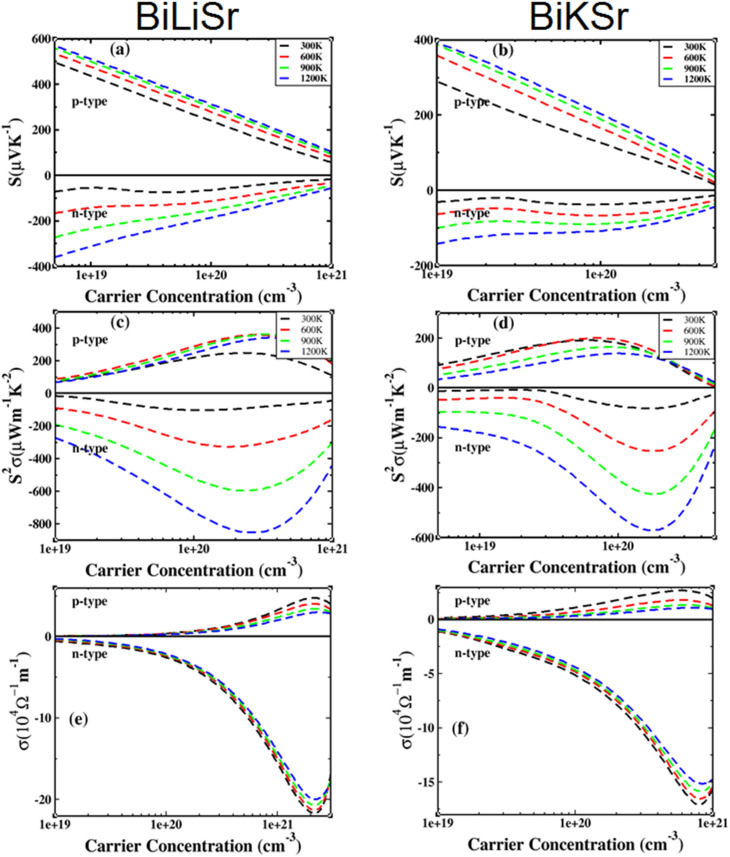
TE properties of BiXSr (X = Li and K) as a function of carrier concentration at different temperatures: (a and b) Seebeck coefficient, (c and d) power factor, and (e and f) electrical conductivity.

Fig. 4(a) and (b) show the variation of the Seebeck coefficient (*S*) as a function of carrier concentration ranging from 1 × 10^19^ to 1 × 10^21^ cm^−3^ at different temperatures (300–1200 K). At a fixed carrier concentration, *S* increased with increasing temperature. If the bipolar effect at high temperatures is neglected, p-type-doped BiXSr (X = Li and K) exhibits higher *S* values than n-type-doped BiXSr at each given temperature. The absolute value of *S*, |*S*|, decreases with increasing carrier concentration, and the |*S*| values for p-type BiXSr (X = Li and K) are significantly higher than those for n-type BiXSr.

In narrow-bandgap semiconductors, a steeper slope of the density of states near the energy gap corresponds to a larger effective mass, which can lead to a higher Seebeck coefficient.^61,62^ The relationship between the effective mass and the Seebeck coefficient is given as follows:14
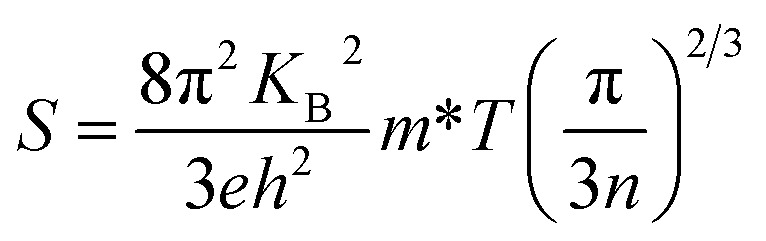
where *K*_B_, *h*, *e*, *T*, *n*, and m* are the Boltzmann constant, Planck constant, electronic charge, absolute temperature, carrier concentration, and effective mass, respectively. At a constant temperature, the Seebeck coefficient depends on the effective mass and the carrier concentration. In combination with the results shown in Fig. 3, the VB of BiXSr (X = Li and K) exhibits a relatively flat and dispersed band structure, whereas the CBM exhibits a sharper band profile. Consequently, the effective mass of holes is higher than that of electrons, indicating that the TE performance of p-type BiXSr (X = Li and K) is superior to that of n-type BiXSr.

The power factor (PF) is a crucial parameter for measuring thermoelectric performance. Fig. 4(c) and (d) show the variation of the power factor with carrier concentration for BiXSr (X = Li and K) compounds at different temperatures. At each fixed temperature, the power factor initially increases to a peak value and then gradually decreases with increasing carrier concentration. At each fixed concentration, the power factor increases with temperature. The estimates indicate that the optimal carrier concentration for n-type compounds is higher than that for p-type materials.

The optimal PF values for p- and n-type BiLiSr compounds are 364.15 µW m^−1^ K^−2^ (carrier concentration of 3.33 × 10^20^ cm^−3^) and 852.14 µW m^−1^ K^−2^ (carrier concentration of 2.74 × 10^20^ cm^−3^), respectively. The corresponding values for BiKSr are 200 µW m^−1^ K^−2^ (carrier concentration of 7.34 × 10^19^ cm^−3^) and 572.85 µW m^−1^ K^−2^ (carrier concentration of 1.80 × 10^20^ cm^−3^), respectively. A comparison of the PF values reveals that n-type BiLiSr exhibits relatively higher values than p-type BiLiSr, suggesting that n-type BiLiSr compounds can demonstrate superior TE performance.

The electrical conductivities of the BiXSr (X = Li and K) compounds are shown in Fig. 4(e) and (f). The *σ* values for both n-type and p-type materials initially increased and then decreased with increasing carrier concentration. Meanwhile, the electrical conductivity exhibits a weak dependence on temperature. The *σ* values of n-type materials are higher than those of p-type materials.

**Fig. 5:**
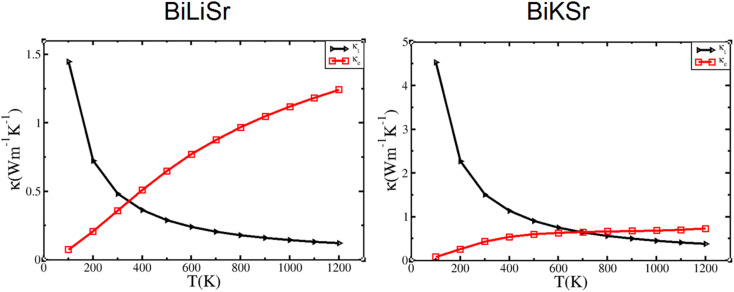
Variation in the lattice and electronic thermal conductivities of BiXSr (X = Li and K) with temperature.

Thermal conductivity, a key factor affecting the efficiency of TE materials, comprises electronic (*κ*_e_) and lattice (*κ*_l_) components, related by the following expression:

15 *κ* = *κ*_e_ + *κ*_l_

where *κ*_e_ represents the electronic contribution and *κ*_l_ represents the phononic (lattice vibrational) contribution. The electronic thermal conductivity *κ*_e_, which depends on carrier concentration and temperature, was determined using the BoltzTraP code. The lattice thermal conductivity *κ*_l_ was calculated using the Callaway model, as shown in eqn (2). Using the Slack's equation, the temperature dependence of the lattice thermal conductivity was calculated and plotted, as shown in Fig. 5. The electronic thermal conductivity *κ*_e_ as a function of temperature was calculated according to the Wiedemann–Franz law, as follows:

16 *κ*_e_ = *LσT*

where *L* is the Lorentz number. The results are plotted and shown in Fig. 5. It can be observed that the lattice thermal conductivity of BiXSr (X = Li and K) decreases with increasing temperature. At 300 K, the lattice thermal conductivities of BiLiSr and BiKSr are 0.48 and 1.48 W m^−1^ K^−1^, respectively. The calculated result for BiKSr is in good agreement with the values reported in previous theoretical studies of 1.45 W m^−1^ K^−1^ (ref. 63) and 1.96 W m^−1^ K^−1^.^31^ In contrast, the calculated value for BiLiSr is significantly lower than the previously reported theoretical values of 2.48 W m^−1^ K^−1^ (ref. 63) and 3.04 W m^−1^ K^−1^.^31^

When the temperature exceeds 300 K, the lattice thermal conductivity changes more slowly, and the curve becomes smooth, indicating that the BiXSr (X = Li and K) compounds exhibit a good TE response. However, the electronic thermal conductivity increases with increasing temperature. The variation curve for BiKSr is relatively flatter than that for BiLiSr, showing little changes throughout the high-temperature range. The lattice thermal conductivity is equal to the electronic thermal conductivity at specific temperatures. The specific temperatures are 347 and 683 K for BiLiSr and BiKSr, respectively. When the temperature exceeds these specific values, the thermal conductivity is primarily attributed to electronic thermal conductivity. A similar phenomenon has been observed for TiNiSn.^64^

**Fig. 6:**
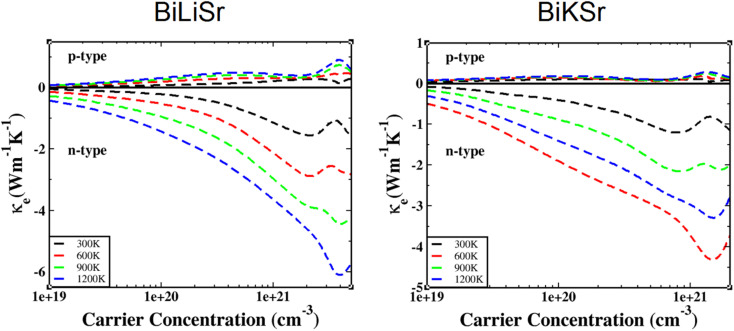
Variation in electronic thermal conductivity of BiXSr (X = Li and K) as a function of carrier concentration at different temperatures.

As shown in Fig. 6, the relationship between the electronic thermal conductivity *κ*_e_ of BiXSr (X = Li and K) and the carrier concentration was investigated at different temperatures. The variation of electronic thermal conductivity with temperature follows a similar trend for both materials, and n-type materials are superior to p-type materials. For both n- and p-type materials, the electronic thermal conductivity increases with increasing temperature, a phenomenon observed in previous studies.^34,45^ At a constant temperature, the electronic thermal conductivity of n-type materials increases with carrier concentration, peaking at approximately 1.5 × 10^21^ cm^−3^, and then decreases.

The performance of TE materials in practical applications is determined by the dimensionless figure of merit *ZT*, which is defined as *ZT* = *ZT*_e_ × *κ*_e_/(*κ*_e_ + *κ*_l_).^65^ At high temperatures, the increase in thermally excited electron–hole pairs leads to an enhanced contribution of electrons to the thermal conductivity. Additionally, a decrease in the phonon mean free path (*L*_ph_) results in a significant reduction in lattice thermal conductivity. Furthermore, as shown in Fig. 5, the thermal conductivity primarily arises from the electronic contributions at elevated temperatures. Therefore, it can be assumed that when the temperature exceeds 600 K, the contribution of lattice thermal conductivity is negligible.

**Fig. 7:**
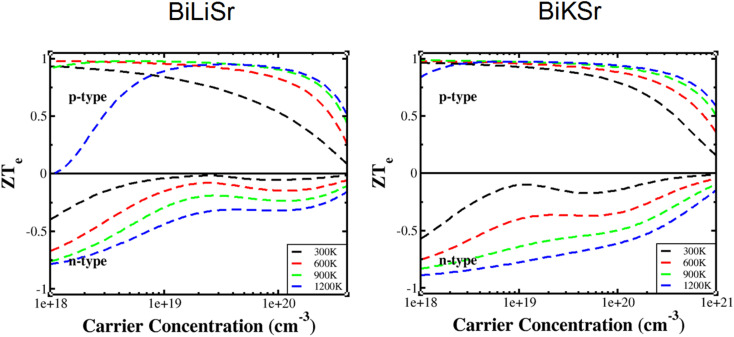
Variation in the *ZT*_e_ ratio of BiXSr (X = Li and K) as a function of carrier concentration at different temperatures.

The *ZT*_e_ = *S*^2^*σT*/*κ*_e_ ratio is independent of the relaxation time *τ*. The variation of the *ZT*_e_ ratio as a function of carrier concentration at temperatures above 600 K is shown in Fig. 7. If the bipolar effect is neglected, *ZT*_e_ increases with increasing temperature at a fixed carrier concentration, and *ZT*_e_ decreases with increasing carrier concentration at a fixed temperature. For BiXSr (X = Li and K), the maximum *ZT*_e_ values are achieved within a carrier concentration range of approximately 1.5 × 10^18^ to 9.5 × 10^18^ cm^−3^. Overall, the *ZT*_e_ values for p-type materials are significantly higher than those for n-type materials. Both BiLiSr and BiKSr reach a maximum *ZT* value of 0.99 at 900 K. As the influence of lattice thermal conductivity was not considered, the obtained TE figure of merit may be slightly overestimated. However, BiXSr (X = Li and K) performed comparable to many other Heusler alloys in previous studies,^66,67^ making the materials promising candidate for p-type components in TE devices.

## 4. Conclusion

Based on first-principles calculations and Boltzmann transport theory, this study systematically investigates the electronic structure, mechanical properties, and TE performance of ternary half-Heusler compounds BiXSr (X = Li and K). The mechanical parameters and phonon spectra of BiXSr (X = Li and K) were calculated using optimised lattice parameters. The results show that the Poisson's ratios of BiLiSr and BiKSr are 3.484 and 2.037, respectively, indicating that both materials are ductile. No imaginary or negative frequencies are observed in the phonon spectra of BiLiSr or BiKSr, confirming their dynamic stability. Electronic structure calculations reveal that BiLiSr is a direct-bandgap semiconductor, whereas BiKSr is an indirect-bandgap semiconductor. The narrow bandgap of BiKSr suggests its potential for excellent TE performance.

For the first time, this study systematically investigates the variations of parameters such as the Seebeck coefficient, power factor, electronic thermal conductivity, lattice thermal conductivity, and electronic thermoelectric figure of merit (*ZT*_e_) of BiXSr (X = Li and K) as functions of carrier concentration and temperature. The lattice thermal conductivity of BiKSr at room temperature is in good agreement with the results of previous theoretical studies. Furthermore, the *ZT*_e_ values of both BiLiSr and BiKSr approach 0.99 at 900 K, indicating that BiXSr (X = Li and K) alloys are promising TE materials over a wide temperature range.

## Conflicts of interest

There are no conflicts to declare.

## Data Availability

The data generated and analyzed in this study are included within the manuscript. Additional data supporting this work are available from the corresponding author upon reasonable request.
